# Repair of symptomatic bilateral L5 spondylolysis with autogenous iliac crest graft and temporary intersegmental pedicle screw fixation in youth

**DOI:** 10.1186/s13018-021-02534-y

**Published:** 2021-07-02

**Authors:** Zhi-Cheng Zhang, Yang Zhang, Li-Zhi Zhang, Kai Guan, Guang-Min Zhao, Da-Jiang Ren, Fang Li, Tian-Sheng Sun

**Affiliations:** Department of Orthopedic, The Seventh Medical Center of Chinese PLA General Hospital, Beijing, 100700 China

**Keywords:** Lumbar spondylolysis, Repair, Internal fixation, Motion segment

## Abstract

**Background:**

When symptomatic spondylolysis fail to respond to nonoperative treatment, surgical management may be required. A number of techniques have been described for repair by intrasegmental fixation with good results; however, there are still some problems. We reported a repair technique with temporary intersegmental pedicle screw fixation and autogenous iliac crest graft. The aim of present study is to assess the clinical outcomes of L5 symptomatic spondylolysis with this technique.

**Methods:**

A retrospective analysis of 128 patients with L5 spondylolysis treated with this method was performed. According to CT scan, the spondylolysis were classified into 3 categories: line, intermediate, and sclerosis type. The diagnostic block test of L5 bilateral pars defect was done in all patients preoperatively. The sagittal and axial CT images were used to determine the bone union. The healing time, complications, number of spina bifida occulta, Japanese Orthopedic Association (JOA) score, and VAS for back pain were recorded. After fixation removal, the rate of ROM preservation at L5S1 was calculated.

**Results:**

There were 97 patients (194 pars) followed with mean follow-up of 23 months (range, 12–36 months). The union rate of pars was 82.0% at 12 months and 94.3% at 24 months postoperatively. Low back pain VAS significantly (P < 0.05) improved from preoperative mean value of 7.2 to 1.3 at the final follow-up postoperatively (P < 0.05). JOA score increased significantly postoperatively (P < 0.05) with average improvement rate of 79.3%. The rates of L5S1 ROM preservation were 79.8% and 64.0% after fixation removal at 1 and 2 years postoperatively. There were 3 patients of delayed incision healing without other complications.

**Conclusions:**

Although sacrificing L5S1 segment motion temporarily, more stability was obtained with intersegmental fixation. This technique is reliable for spondylolysis repair which has satisfactory symptom relief, high healing rate, low incidence of complications, and preserve a large part of ROM for fixed segment.

## Background

Lumbar spondylolysis is a bony defect in the pars interarticularis. The current theory believes fatigue or stress fracture of pars with developmental factors is the most widely accepted reason for the occurrence of isthmic defect [1]. Previous studies showed repetitive lumbar extension and rotation were related to the development of spondylolysis [2]. Most of spondylolysis are found at L5 level. The condition is often asymptomatic but may be the cause of low back pain (LBP) in young adults. One fourth of individuals with spondylolysis may suffer from LBP. A study in Japan described 70% of patients with bilateral pars defects are associated with varying degrees of vertebral spondylolisthesis, and some cases need surgery [3]. Symptomatic spondylolysis should be first treated by nonoperative care, despite several operative options available. When they fail to respond to conservative treatment, surgical interventions may be required. The aims of surgery are to reduce pain, stabilize affected segment, promote healing of pars defect, and control spondylolisthesis development effectively.

Surgical methods for lumbar spondylolysis could be roughly divided into two categories, fusion and repair. Meaningless sacrifice of fairly normal disc and segmental motion make stricter indication for fusion [4–7]. The concept of repair surgery is similar to osteosynthesis. The points of surgery are pars defect debridement, bone graft, and local stabilization. A number of techniques had been described for repair by intrasegmental fixation with good results. Implanting a screw across the par is technically difficult with high incidence of screw fracture. Furthermore, the screw occupies the bone graft area for fusion [8, 9]. Wiring techniques have the disadvantages of more bleeding, wire break, and insufficient stabilization. Pedicle screw-hook or U rod methods are able to provide more stability; however, the L5 lamina often has developmental abnormalities which make the pedicle screw and hook too close to have enough room for tightening the link to compress the bone graft in the defect. In fact, these intrasegmental fixation techniques abovementioned cannot be stronger than intersegmental pedicle screws in the control of low lumbar extension and rotation stress. In particular, the pars interarticularis of L5 is sheared during extension and rotation by the inferior articular process of L4 and the superior articular process of the sacrum acting as a pair of wedges. In this theory, L5S1 pedicle screws can achieve higher healing rate and stability of L5 pars defect, especially for patients with spina bifida occulta (SBO), large gap of par, grade I spondylolisthesis, more sagittal segmental angular motion, and L5S1 disc degeneration. If most of the segmental motion is preserved after removing the temporary pedicle screws for healed patients, then it is a good method which has same philosophy with thoracolumbar fracture pedicle fixation and removal. After all, the key point for treatment is how to establish the association between the symptoms and pars defect [10]. The diagnostic block tests should be performed to determine the pars defect is the cause of pain.

We describe a direct repair technique with temporary intersegmental pedicle screw fixation and autogenous iliac crest graft. The purpose of this retrospective case series study is to evaluate the clinical outcomes of L5 symptomatic bilateral lumbar spondylolysis with this method after pars block test.

## Materials and methods

### Study population

**Fig. 1:**
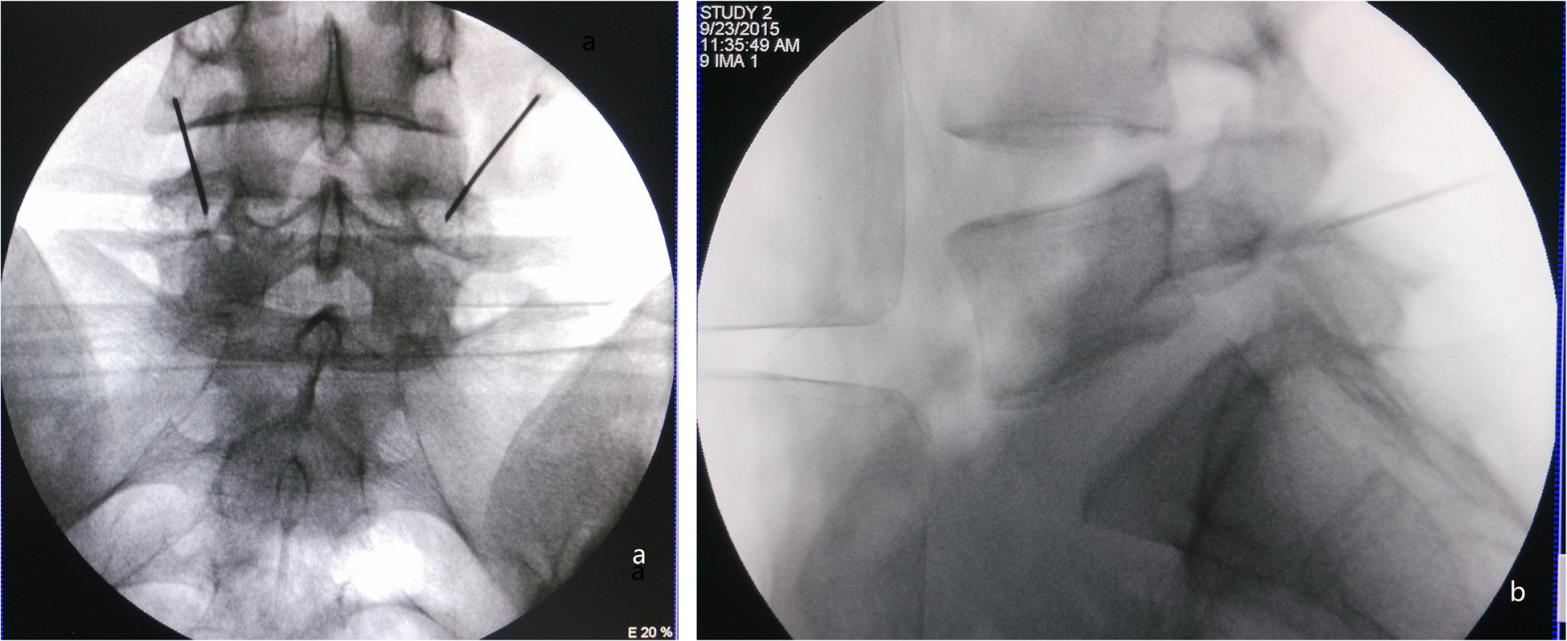
The diagnostic block test of L5 bilateral pars defect. A standard anteroposterior (**a**) and lateral view (**b**) a of C-arm fluoroscope

Between October 2013 and October 2016, 128 consecutive L5 spondylolysis patients were included in this retrospective study. All of them met the following inclusive criteria: (1) age range (18–36 years); (2) LBP VAS ≥ 5.0 (without radiculopathy); (3) lack of response to conservative treatment for 6 months, including medication and brace; (4) bilateral spondylolysis and no more than I grade spondylolisthesis; and (5) more than 50% pain relief by bilateral pars gap block test (Fig. 1).

And the exclusive criteria are as follows: (1) multi-segmental spondylolysis (≥ 2 levels) and (2) Pfirrmann III-V grade disc degeneration [11, 12] for L5S1 and other lumbar segments. Finally, only 97 cases data were collected completely (92 men and 5 women; mean age 22.5 ± 4.5 years). Eight-seven patients had a clear history of repeated back extension and rotation motions. Fifty-eight cases had experience of transient radiation leg pain.

### Operative procedures

The patient was placed in the prone position under general anesthesia. A midline incision was performed, the bilateral muscles were stripped off the spinous process, lamina, and the base of the transverse process carefully protecting facet joint capsule intact. The defects were exposed. The entry point of the L5 pedicle screw should be selected as cephalic as possible to provide more bone graft space for the defect. After L5S1 pedicle screws implantation, the fibrous scar tissue was removed completely. The pars defect, corresponding lamina, and transverse process base were decorticated to bleeding with burr. The iliac crest cylindrical bone mass was taken with a circular saw within posterior superior iliac spine leaving the inner and outer plate intact in the same incision. Iliac bone was implanted in the gap and impacted bilaterally. The residual bone fragments were placed from the transverse process to the lamina covering the defect. The appropriate rods were contoured and connected with pedicle screws. When tightening the screw, the defects and bone graft were compressed. The negative drainage was placed and the incision was closed. The lumbar brace was used for 3 months after surgery. When bilateral pars defects union, pedicle screw should be removed through the midline incision and intermuscular approach.

### Evaluation of clinical outcomes

**Fig. 2:**
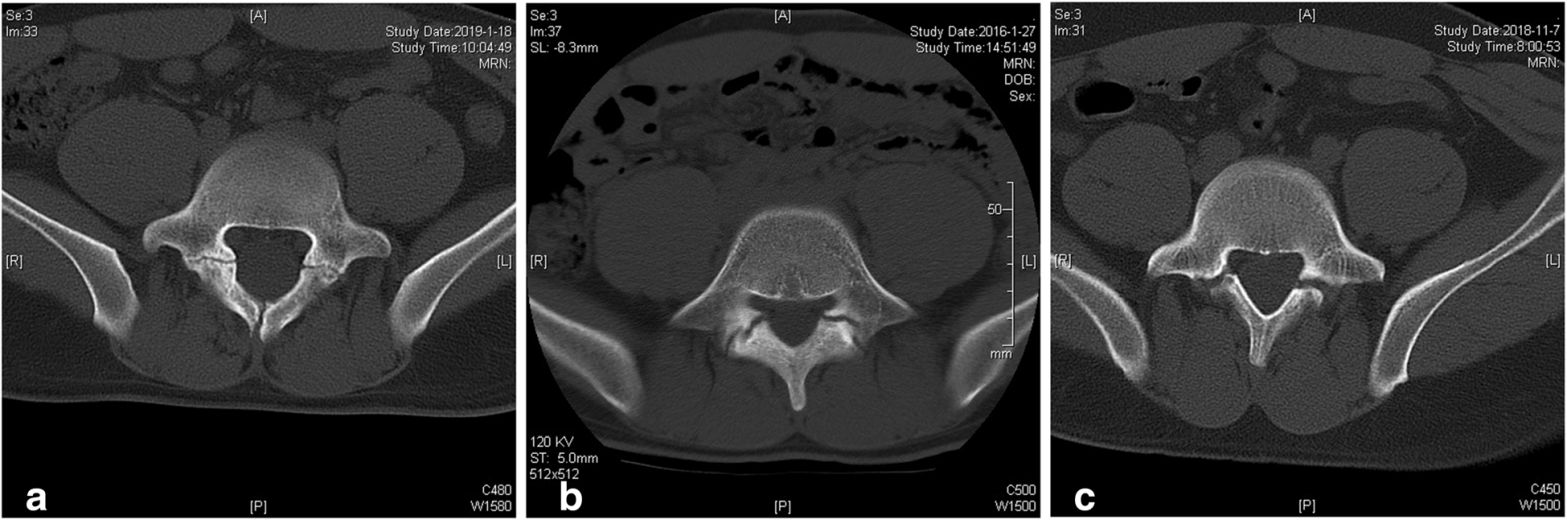
CT classification of lumbar spondylolysis. **a** Line type, **b** intermediate type, and c sclerosis type

All patients should take anteroposterior, lateral, dynamic, double oblique radiography, 3D CT scan, and lumbar MRI preoperatively. The diagnosis was established according to the CT results. Based on the axial CT scan [13], the classification was shown in Fig. 2. In type I (line type), gap is very narrow, similar to hairline; in type II (intermediate type), show a clear bone gap, no atrophy and sclerosis edge of bony defect; and in type III (sclerosis type), enlarged bony gap with manifestations of bone atrophy and sclerosis. L5S1 disc was evaluated by lumbar MRI with Pfirrmann classification. The number of patients with spina bifida occulta was calculated. Postoperatively, X-rays were taken regularly. According to the follow-up plan, first postoperative CT was taken at 6 months after surgery. Bone union was defined as a bony continuity at the pars defect in axial and sagittal CT scan. When the bone defect or a clear zone exists, the nonunion was defined. The indication for removing internal fixation was bilateral defects union. After fixation removal, lumbar spine flexion-extension X-rays should be taken to evaluate the ROM of the fixed segment.

The surgical data of operating time, blood loss, and complications were noted. The LBP and functional outcomes assessment were carried out using the Japanese Orthopedic Association (JOA) and VAS with regular follow-up plan. The radiographic examination and functional evaluation were followed to 3 months after removal of internal fixation. The ROM of L5S1 was measured before repair and after fixation removal.

The time of healing was recorded. The union rate was calculated at different follow-up time according to different reference. All quantitative data were described as mean ± standard deviation. The Student t-test and ANOVA method were undertaken. The difference was statistically significant with P < 0.05.

## Results

Thirty-one of the patients were lost to follow-up. Night-seven patients data were collected completely with the mean follow-up of 23 months (range, 12–36 months). The average operation time was 121 ± 31 min (range, 90–180 min) with a mean blood loss of 100 ± 21 ml (range, 50–200 ml). On the basis of mentioned CT classification, there were 36, 126, and 32 pars in the line, intermediate, and sclerosis type, respectively.

### Bony union

At 6 months after operation, there were 41 pars healed (pars union rate 21.1%), and 17 patients healed bilaterally (patient union rate 17.5%). Ninety-four pars healed (48.5%), 34 patients bilaterally (35%) at 9 months; 159 pars healed (82.0%), 72 patients bilaterally (74.2%) at 12 months; and 183 pars healed (94.3%), 90 patients bilaterally (92.8%) at 24 months (Table 1). The pars union rate for CT classification were 86.1% (type I), 7.1% (type II), and 3.1% (type III) at 6 months; 100.0% (type I), 43.7% (type II), and 9.4% (type III) at 9 months; 84.1% (type II) and 53.1% (type III) at 12 months; 96.8% (type II) and 78.1% (type III) at 24 months after surgery (Table 2). The union rates of pars and patients with spina bifida occulta were 78.9% and 68.4% at 12 months and 92.1% and 84.2% at 24 months, respectively (Table 3). At this follow-up, there were still 7 nonunion patients of which 3 type II (1 bilateral, 2 unilateral) and 4 type III (3 bilateral, 1 unilateral). For these nonunion patients, “wait and see” strategy was carried out. There were 3 unilateral nonunion patients and one bilateral patients (type II) healed in three years postoperatively. For the other 3 nonunion patients, S1 pedicle screws were removed; second graft, rhBMP-2, and U-shape rod technique were performed in revision surgery. The L5 pars of the three patients all healed bilaterally 12 month, 15 months, and 18 months after the revision operation.

**Table 1:** Union rate of spondylolysis

Time (postop)	Number of healed pars	Union rate of pars	Number of healed patients	Union rate of patients
6 months	41	21.1%	17	17.5%
9 months	94	48.5%	34	35.1%
12 months	159	82.0%	72	74.2%
24 months	183	94.3%	90	92.8%

**Table 2:** Union rate of spondylolysis with reference to CT classification

Time (postop)	Type I	Type II	Type III
6 months	86.1% (31/36)	7.1% (9/126)	3.1% (1/32)
9 months	100.0% (36/36)	43.7% (55/126)	9.4% (3/32)
12 months	100.0% (36/36)	84.1% (106/126)	53.1% (17/32)
24 months	100.0% (36/36)	96.8% (122/126)	78.1% (25/32)

**Table 3:** Union rate of spina bifida occulta

Time (postop)	Union rate of pars	Union rate of patients
6 months	15.8% (6/38)	10.5% (2/19)
9 months	47.3% (18/38)	26.3% (5/19)
12 months	78.9% (30/38)	68.4% (13/19)
24 months	92.1% (35/38)	84.2% (16/19)

### Comparison of VAS and JOA scores before and after surgery

Compared with preoperative levels, the mean VAS score of LBP improved from 7.2 ± 2.1 to 1.3 ± 0.4 at the final follow-up (P < 0.05, Table 4). The mean JOA score increased from an initial score of 20.8 ± 3.5 preoperatively to 27.3 ± 1.2 at the latest follow-up (P < 0.05, Table 4). The mean JOA improvement rate was 79.3%.

**Table 4:** Comparison of VAS and JOA scores

Scores	Pre-op	Last follow-up	T value	P value
VAS	7.2 ± 2.1	1.3 ± 0.4	27.634	0.0000
JOA	20.8 ± 3.5	27.3 ± 1.2	− 17.302	0.0000

*VAS* visual analog scale, *JOA* Japanese Orthopedic Association

### Complications

No intraoperative complications occurred such as cerebrospinal fluid leakage and nerve injury. No postoperative infection complications were found. There were three cases of delayed incision healing.

### ROM of fixed segment

Preoperatively, the ROM of L5S1 was 8.9° ± 4.1° (2.7–17.2°). The internal fixation of 60 patients and 15 patients with bilateral union were removed at 12 months and 24 months postoperatively. The dynamic lumbar lateral radiograph was taken at 3 months after removal with the L5S1 sagittal ROM of 7.1° ± 3.7° (2.0–14.5°) for 12 months and 5.7° ± 2.7° (2.0–14.0°) for 24 months, respectively. The rate of motion preservation is 79.8% and 64.0% after fixation removal at 1 and 2 years postoperatively.

## Discussion

Previous studies had found that the most common lumbar spondylosis involved L5 level [14, 15]. It almost always occurs bilaterally at L5. The pathogenesis of lumbar spondylolysis and why the L5 is the most frequently involved level are still controversial. But the most popular explanation is that an underlying dysplastic pars interarticularis make it susceptible to the repetitive extension and/or rotation activities resulting in a fatigue or stress fracture [16–18]. Furthermore, the L5 locates at the junctional region between mobile lordotic lumbar spine and fixed kyphotic sacrum that indicating the greatest static and dynamic stress resulted from daily activities. Additionally, the pars of L5 are pinched by L4 inferior articular process and S1 superior articular process during hyperextension that is called the classic “pincer” theory. When hyperextension, the L5 pars have to bear more shearing stress from impaction of L4 and S1 adjacent articular process. The lumbosacral-pelvic parameters and morphology may be the contribution to the high involvement of L5.

Although lumbar spondylolysis accounts for around 6% of the general population [3], most of these people are asymptomatic [19]. However, it is reported to be the common cause of LBP in young person who like sports [20, 21]. Several studies had analyzed the association of the symptoms and spondylolysis with radiologic method. Inflammatory action of pars defect and adjacent pedicle with edema or fluid signals play the important roles in the occurrence of LBP. The diagnostic block test of pars defect has been recommended to determine the relationship between spondylosis and LBP in several literatures [22, 23]; however, no specific study to evaluate the diagnostic value. Undoubtedly, therapeutic interventions should aim at pain relief first and union of defect secondly. Consequently, in our study, the block test was done before operation. Unfortunately, we cannot provide enough scientific evidence for 50% pain relief rate as the threshold connecting LBP with spondylolysis. The mild disc degeneration (Pfirrmann I-II) patients were included in this study which may be the main cause for 50% threshold. In fact, more elaborate work should be performed to establish the threshold in spite of many difficulties. Fortunately, in this study, the VAS and JOA of patients improved significantly according to case inclusive criteria of 50% response to pars defect lidocaine block. Primarily, pars defect diagnostic block test may be a kind of prediction of successful pain relief following pars repair. Moreover, the pain from disc degeneration may decrease by intersegmental pedicle screw fixation. Autogenous iliac crest bone graft is the gold standard of bone grafting for pars union. Unfortunately, there is a frequent incidence of persistent donor site pain after harvest. For this reason, the iliac crest bone was harvested in posterior superior iliac spine by a circular saw leaving the inner and outer plate intact without new incision. It is like making a hole in iliac crest with least exposure. Good outcomes of LBP VAS indicated the bone harvest method is successful.

**Fig. 3:**
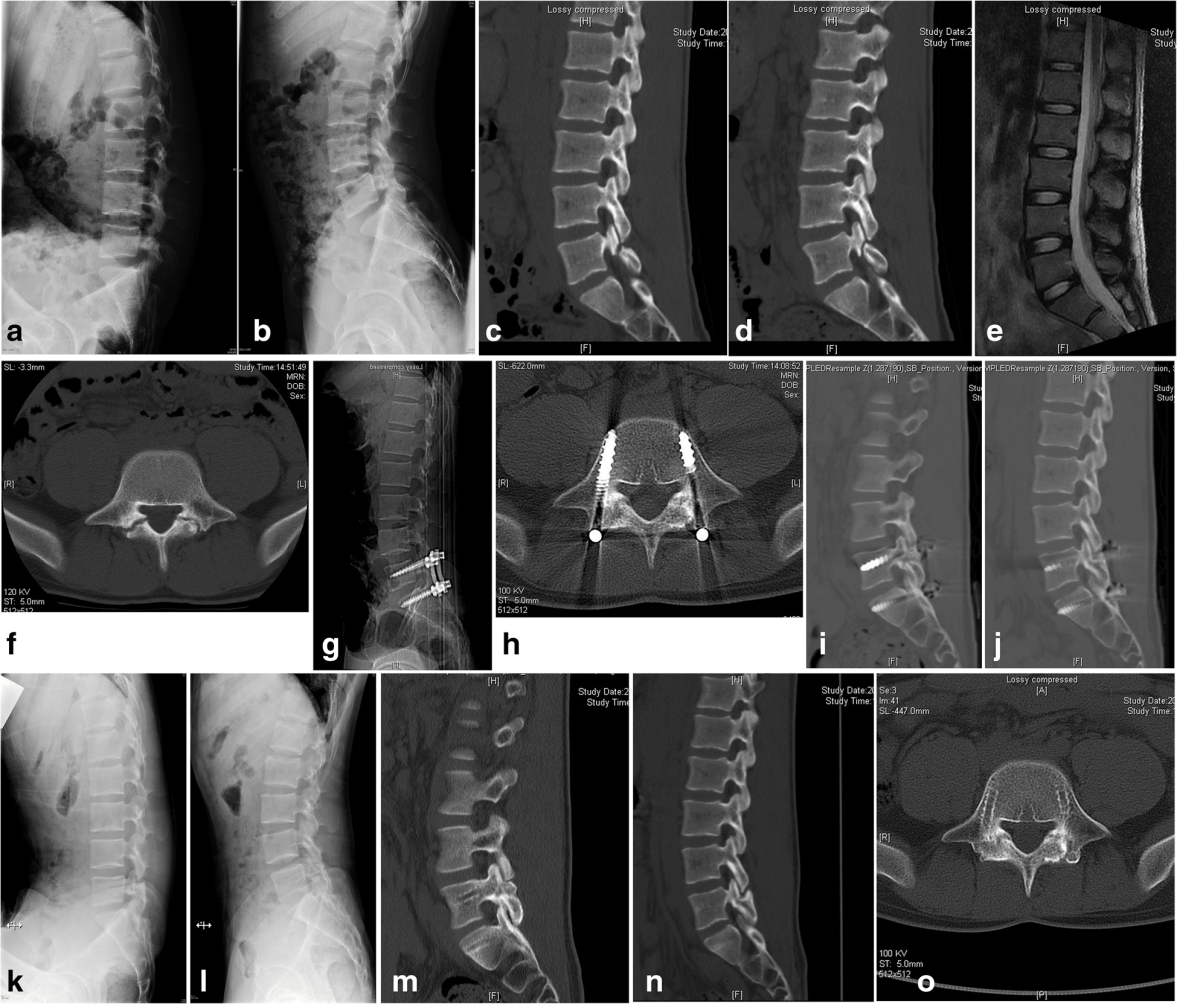
A young male patient of symptomatic bilateral spondylolysis repaired by autogenous iliac crest graft and temporary intersegmental pedicle screw fixation. **a**–**c** Preoperative CT scan showed L5 bilateral pars defect (CT classification Type II). **d** The signals of lumbar intervertebral discs were normal in T2-weighted sagittal MRI. **e**, **f** The ROM of L5S1 segment was 16.2° in dynamic lateral X-ray. **g** Lateral X-ray after surgery. **h**–**j** Bony continuity at the pars defect in axial and sagittal CT scan 12 months postoperatively. **k**, **l** There was 12.9° ROM of L5S1 segment with 79.6% motion preservation at 1 year after surgery. **m**–**o** Solid bone union showed in CT scan after fixation removal

It is no accident that the most controversial point of this technique is temporary fixation of L5S1 motion segment with intersegment pedicle screws. That usually means a degree of segmental motion loss even though temporary stabilization. However, what are the advantages of intersegment fixation? Although there were no biomechanical studies to compare the intersegmental pedicle screw with main intrasegmental stabilization technologies, logically, as the three-column spinal fixation, the biomechanical performances of pedicle screws should be better in control of intersegmental extension and rotation stress [24]. A very interesting clinical and biomechanical study showed spondylolysis originates in the ventral aspect of the pars interarticularis (Fig. 3c, d) just because the higher stress was found at the caudal-ventral aspect in all loading modes when repeated hyperextension and rotation activity [25]. That conclusion reminded us of the design flaws of intrasegmental fixation even though these techniques had good performances in clinical application and biomechanical tests. As the most commonly used technologies, the screw-hook and screw-wire technique are placed on the dorsal aspect of pars interarticularis, when tightening the system, the moment of force is behind the lamina and pars. Therefore, we made such a hypothesis that the two methods could not be stronger than pedicle screw in reduction of ventral stress when hyperextension and rotation. Of course, the postulate needs to be clarified by further biomechanical research. In some of our cases, including a relatively large gap of par, grade I spondylolisthesis, translation, and more sagittal segmental angulation, intrasegmental techniques are insufficient for restorage, stabilization, and maintenance of the relationship of pars defect and L5S1 segment. It is very important for healing of spondylolysis and symptoms recovery. The union rate of this method was higher than those described in previous reports [4]. With reference of CT classification, all the type I cases healed at 9 months, but the slowest healing occurred in type III cases. Interestingly, there are few reports about surgical management of spondylolysis with SBO, spinous process, and lamina dysplasia that cannot become a strong anchor point. In the study of segmental wire fixation for spondylolysis associated with SBO [26], two of four SBO cases showed no union bilaterally. Our data showed relatively high healing rate of spina bifida occulta cases which is better than previous. Therefore, the CT classification III and spina bifida occulta are the risk factors of nonunion. In this study, 79.8% of segmental ROM for healed patients 1 year postoperatively, 64.0% for 2 years, were preserved. Our findings described the longer fixed time, the greater loss of ROM. Accordingly, further research should be focused on the union promoting factors to get healing and remove fixation as soon as possible.

There are some limitations to this study. First, because of the retrospective nature of this study, the strength of evidence was not very high in evaluating outcomes of pain and function. Second, part of patients was lost to follow-up which may result in exclusive bias. Third, there were no control group, and the study might have been subjected to intervention biases. Although the size of the sample was big, it was still a single-center study.

## Conclusion

In conclusion, although the L5S1 motion segment was fixed temporarily with intersegmental pedicle screw, the segment obtained more stability to decrease hyperextension and rotation stress of L5 pars and improve the union rate, especially for patients of CT classification III, grade I spondylolisthesis, mild disc degeneration, and spina bifida occulta. The diagnostic par block test should be recommended in the repair of spondylolysis which is an available link between symptoms and pars defect. The gratifying result was that a large part of segmental motion was preserved. This technique is reliable for lumbar spondylolysis repair which has satisfactory symptom relief, high healing rate, and low incidence of complications.

## Data Availability

The datasets collected in this study are not publicly available because of the special nature of our hospital in Army. A further study about lumbar spondylolysis is in progress, if necessary, the data are available from the corresponding author on reasonable request under permission and supervision.

## Abbreviations

CT: Computed tomography
LBP: Low back pain
JOA: Japanese Orthopedic Association
VAS: Visual analog scale
SBO: Spina bifida occulta
MRI: Magnetic resonance imaging
ANOVA: Analysis of variance
rhBMP-2: Recombinant Human Bone Morphogenetic Protein-2

## References

[1] Leone A, Cianfoni A, Cerase A, Magarelli N, Bonomo L (2011). Lumbar spondylolysis: a review. Skeletal Radiol..

[2] Goetzinger S, Courtney S, Yee K, Welz M, Kalani M, Neal M. Spondylolysis in young athletes: an overview emphasizing nonoperative management. J Sports Med (Hindawi Publ Corp). 2020;2020:9235958. 10.1155/2020/9235958. PMID: 32047822; PMCID: PMC7001669 10.1155/2020/9235958 PMC7001669 32047822

[3] Sakai T, Sairyo K, Takao S, Nishitani H, Yasui N (2009). Incidence of lumbar spondylolysis in the general population in Japan based on multidetector computed tomography scans from two thousand subjects. Spine..

[4] Mohammed N, Patra DP, Narayan V, Savardekar AR, Dossani RH, Bollam P, Bir S, Nanda A (2018). A comparison of the techniques of direct pars interarticularis repairs for spondylolysis and low-grade spondylolisthesis: a meta-analysis. Neurosurg Focus..

[5] Gagnet P, Kern K, Andrews K, Elgafy H, Ebraheim N (2018). Spondylolysis and spondylolisthesis: a review of the literature. J Orthop..

[6] Randall RM, Silverstein M, Goodwin R (2016). Review of pediatric spondylolysis and spondylolisthesis. Sports Med Arthrosc Rev..

[7] Pizzutillo PD, Mirenda W, MacEwen GD (1986). Posterolateral fusion for spondylolisthesis in adolescence. J Pediatr Orthop.

[8] Giudici F, Minoia L, Archetti M, Corriero AS, Zagra A (2011). Long-term results of the direct repair of spondylolisthesis. Eur Spine J.

[9] Sairyo K, Goel VK, Faizan A, Vadapalli S, Biyani S, Ebraheim N (2006). Buck’s direct repair of lumbar spondylolysis restores disc stresses at the involved and adjacent levels. Clin Biomech (Bristol, Avon).

[10] Hu SS, Tribus CB, Diab M, Ghanayem AJ (2008). Spondylolisthesis and spondylolysis. J Bone Joint Surg Am..

[11] Urrutia J, Besa P, Campos M, Cikutovic P, Cabezon M, Molina M, Cruz JP (2016). The Pfirrmann classification of lumbar intervertebral disc degeneration: an independent inter- and intra-observer agreement assessment. Eur Spine J..

[12] Castro-Mateos I, Hua R, Pozo JM, Lazary A, Frangi AF (2016). Intervertebral disc classification by its degree of degeneration from T2-weighted magnetic resonance images. Eur Spine J..

[13] Sairyo K, Sakai T, Yasui N, Dezawa A (2012). Conservative treatment for pediatric lumbar spondylolysis to achieve bone healing using a hard brace: what type and how long? Clinical article. J Neurosurg Spine.

[14] Laurent LE, Osterman K (1969). Spondylolisthesis in children and adolescents: a study of 173 cases. Acta Orthop Belg..

[15] Grogan JP, Hemminghytt S, Williams AL, Carrera GF, Haughton VM (1982). Spondylolysis studied with computed tomography. Radiology..

[16] Wiltse LL, Widell EH, Jackson DW (1975). Fatigue fracture: the basic lesion is inthmic spondylolisthesis. J Bone Joint Surg Am..

[17] Sairyo K, Katoh S, Sasa T, Yasui N, Goel VK, Vadapalli S, Masuda A, Biyani A, Ebraheim N (2005). Athletes with unilateral spondylolysis are at risk of stress fracture at the contralateral pedicle and pars interarticularis: a clinical and biomechanical study. Am J Sports Med..

[18] Sairyo K, Katoh S, Sakamaki T, Komatsubara S, Endo K, Yasui N (2003). Three successive stress fractures at the same vertebral level in an adolescent baseball player. Am J Sports Med..

[19] Beutler WJ, Fredrickson BE, Murtland A, Sweeney CA, Grant WD, Baker D (2003). The natural history of spondylolysis and spondylolisthesis: 45-year follow-up evaluation. Spine..

[20] Hefti F, Brunazzi M, Morscher E (1994). Natural course in spondylolysis and spondylolisthesis. Orthopade.

[21] Iwamoto J, Abe H, Tsukimura Y, Wakano K (2004). Relationship between radiographic abnormalities of lumbar spine and incidence of low back pain in high school and college football players: a prospective study. Am J Sports Med..

[22] Suh PB, Esses SI, Kostuik JP (1991). Repair of pars interarticularis defect. The prognostic value of pars infiltration. Spine.

[23] Wu SS, Lee CH, Chen PQ (1999). Operative repair of symptomatic spondylolysis following a positive response to diagnostic pars injection. J Spinal Disord..

[24] Deguchi M, Rapoff AJ, Zdeblick TA (1999). Biomechanical comparison of spondylolysis fixation techniques. Spine..

[25] Terai T, Sairyo K, Goel VK, Ebraheim N, Biyani A, Faizan A, Sakai T, Yasui N (2010). Spondylolysis originates in the ventral aspect of the pars interarticularis: a clinical and biomechanical study. J Bone Joint Surg Br.

[26] Yamamoto T, Iinuma N, Miyamoto K, Sugiyama S, Nozawa S, Hosoe H, Shimizu K (2008). Segmental wire fixation for lumbar spondylolysis associated with spina bifida occulta. Arch Orthop Trauma Surg..

